# APP PROMOTING NONPHARMACOLOGICAL INTERVENTIONS FOR PEOPLE LIVING WITH DEMENTIA IN NURSING HOMES

**DOI:** 10.1093/geroni/igae098.3468

**Published:** 2024-12-31

**Authors:** Minhee Yang, Sinwoo Hwang, EunKyo Kim, Jungwon Cho, Eunhee Cho

**Affiliations:** Yonsei University, Seoul, Republic of Korea; Yonsei University, Seoul, Republic of Korea; Yonsei University, Seoul, Republic of Korea; Yonsei University, Seoul, Republic of Korea; Yonsei University, Seoul, Republic of Korea

## Abstract

Mobile app-based interventions have emerged as a promising approach for providing individualized non-pharmacological interventions to people living with dementia (PLWD) to manage behavioral and psychological symptoms of dementia (BPSD). Offering such interventions can be challenging in nursing homes because of the lack of support from family members and heavy workload when composing and implementing individualized non-pharmacological interventions. This study aimed to develop and evaluate a decision support application for nurses to facilitate non-pharmacological interventions. The research team developed an app that provides symptom-specific non-pharmacological interventions, and experts provided feedback on the app’s usability. Finally, the app was tested with four dyads of PLWD and nurses for four weeks (two weeks of the intervention period only) and was assessed for its overall quality and usability using questionnaires and interviews. Nurses recorded BPSD diaries ranging from 17 to 78 for four weeks. The implementation of symptom-specific, individualized non-pharmacological interventions using the application led to a reduction in BPSD. The nurses most frequently provided individualized music programs, followed by reminiscence programs. The engagement level of each dyad ranged from 3.1 to 3.6 out of 5. Overall, the app was perceived as beneficial in facilitating symptom-specific, individualized non-pharmacological care in nursing homes. Collectively, this study highlights the usefulness of the app in nursing homes by assisting nurses in their decisions regarding non-pharmacological interventions. However, further enhancements are warranted to improve usability and efficacy within the context of nursing homes.

